# An open-label, phase 1 study of androgen receptor antagonist, apalutamide in Japanese patients with metastatic castration-resistant prostate cancer

**DOI:** 10.1007/s10147-019-01526-7

**Published:** 2019-08-24

**Authors:** Tomohiro Tsuchiya, Keiichiro Imanaka, Yuki Iwaki, Ryo Oyama, Katsuyoshi Hashine, Akito Yamaguchi, Hiroji Uemura

**Affiliations:** 1 Gifu University Hospital, Gifu, Japan; 2 Janssen Pharmaceutical K.K., Tokyo, Japan; 3 National Hospital Organization Shikoku Cancer Centre, Matsuyama, Japan; 4 Harasanshin Hospital, Fukuoka, Japan; 5 Yokohama City University Medical Centre, 4-57, Urafune-cho, Minami-ku, Yokohama, 232-0024 Japan

**Keywords:** Androgen receptor antagonist, Apalutamide, Metastatic castration-resistant prostate cancer, Prostate cancer

## Abstract

**Background:**

Apalutamide, a nonsteroidal potent androgen receptor antagonist, was safe and effective in patients with non-metastatic castration-resistant prostate cancer (nmCRPC) and metastatic-CRPC (mCRPC) in global studies. In this phase 1 study, safety, pharmacokinetics (PK), and efficacy of apalutamide were evaluated in Japanese patients with mCRPC.

**Methods:**

In this open-label, multi-center study, patients received apalutamide 240 mg (once-daily, orally) for first 1 week (PK week) during which PK parameters were assessed. 1 week later (Cycle 1 Day1), after reassessing safety, continuous daily dosing (4 weeks/cycle; once-daily orally) was initiated. Endpoints evaluated were: safety, tolerability, PK and antitumour efficacy of apalutamide. Dose-limiting toxicities (DLTs) were evaluated during PK week and Cycle 1.

**Results:**

All six patients received apalutamide. The most common treatment-emergent adverse events (TEAEs) were abdominal discomfort, nasopharyngitis, dysgeusia, rash, and hot flush [2/6 patients (33.3%) each]. No death or DLTs were reported. Grade 3 TEAEs were spinal-cord compression and renal disorder (1/6 patient each). In continuous daily dosing period, PK steady-state of apalutamide was reached approximately by week 4. A significant accumulation of apalutamide was observed (mean accumulation index 3.55), based on AUC_0–24_. Median (range) serum prostate-specific antigen level decreased from 54.42 (8.92–310.11) ng/mL at baseline to 11.70 (0.37–47.74) ng/mL at week 12 with ≥ 50% reduction in 4/6 (66.7%) patients and 90% reduction in 2/6 (33.3%) patients.

**Conclusion:**

Apalutamide had manageable safety profile, without any DLT or any new safety signals, and favourable efficacy in Japanese mCRPC patients. Thus, it was ascertained to be an adequate dosage regimen in Japanese mCRPC patients.

**Trial registration:**

ClinicalTrials.gov identifier: NCT02162836.

**Electronic supplementary material:**

The online version of this article (10.1007/s10147-019-01526-7) contains supplementary material, which is available to authorized users.

## Introduction

Prostate cancer is the 6th leading cause of cancer death in men worldwide (307,000 deaths in 2012) [1]. Prostate cancer is estimated as one of the most common type of cancers in Japanese men and the sixth largest cause of cancer-related death [2–4]. Androgen deprivation therapy remains the mainstay of treatment for advanced castration-sensitive prostate cancer. Despite the effective blocking of androgen biosynthesis and androgen receptor (AR) signalling, most of patients eventually progress and the disease ultimately becomes castration-resistant [5].

Apalutamide, a second generation nonsteroidal potent AR antagonist, selectively binds to the ligand-binding domain of AR with seven–tenfold greater affinity than the clinically approved antiandrogen, bicalutamide. In addition, in contrast to bicalutamide, apalutamide lacks significant AR agonist activity in preclinical models of CRPC and is unable to induce AR nuclear translocation and DNA binding in prostate cancer cells [6]. Apalutamide has a major metabolite JNJ-56142060, a pharmacologically active AR antagonist, with approximately threefold less potency than apalutamide.

In the previous phase 1/2 study [7], apalutamide was safe and well-tolerated at 30–480 mg dose in non-Japanese castration-resistant prostate cancer (CRPC) patients. In the phase 2 part of that study [7], apalutamide at the 240 mg daily dose was safe with promising efficacy [prostate-specific antigen (PSA) response] in patients with non-metastatic-CRPC (nmCRPC) and metastatic-CRPC (mCRPC). The present study was conducted to evaluate the safety, tolerability, pharmacokinetics (PK), and the antitumor effects of apalutamide after a single-dose (240 mg) and multiple-dose administration (240 mg once-daily) of apalutamide in Japanese men with mCRPC.

## Patients and methods

### Patients

Japanese men aged ≥ 20 years with mCRPC who had histologically confirmed adenocarcinoma of the prostate without neuroendocrine differentiation or small cell features were included in this study. Inclusion criteria also involved maintained castrate levels of testosterone [< 50 ng/dL (1.72 nmol/L)] within 4 weeks and serum PSA level of ≥ 2 ng/mL within 2 weeks before enrolment, which had risen on ≥ 2 successive occasions, ≥ 1 week apart. Patients who received first generation antiandrogen as part of an initial combined androgen blockade therapy or as second-line hormonal therapy for continuing disease progression, or who progressed after AR antagonists, 5-α reductase inhibitors, estrogens, and any other anticancer therapy, including chemotherapy given in the adjuvant/neoadjuvant setting were required to be off those medications for ≥ 4 weeks before first dose administration.

The exclusion criteria were a history or current metastases in the brain or untreated spinal-cord compression, progressive epidural disease, prior treatment with the second generation antiandrogens (e.g., enzalutamide), or CYP17 inhibitors (e.g., abiraterone acetate, orteronel, galeterone, and systemic ketoconazole). Patients with a history of seizure or condition that may predispose to seizure, or use of radiopharmaceutical agents (e.g., strontium-89) or investigational immunotherapy (e.g., sipuleucel-T) within 12 weeks or any investigational non-immunologic agent within 4 weeks before the first dose of study agent were also excluded. Patients who used concurrent therapy with medications known to lower the seizure threshold and strong CYP3A4 inhibitors or inducers, herbal, and non-herbal products that could decrease serum PSA level, systemic (oral/IV/IM) corticosteroids, or any other experimental treatment on another clinical trial within 4 weeks before the first dose of study agent were excluded.

### Study design and treatment

**Fig. 1:**
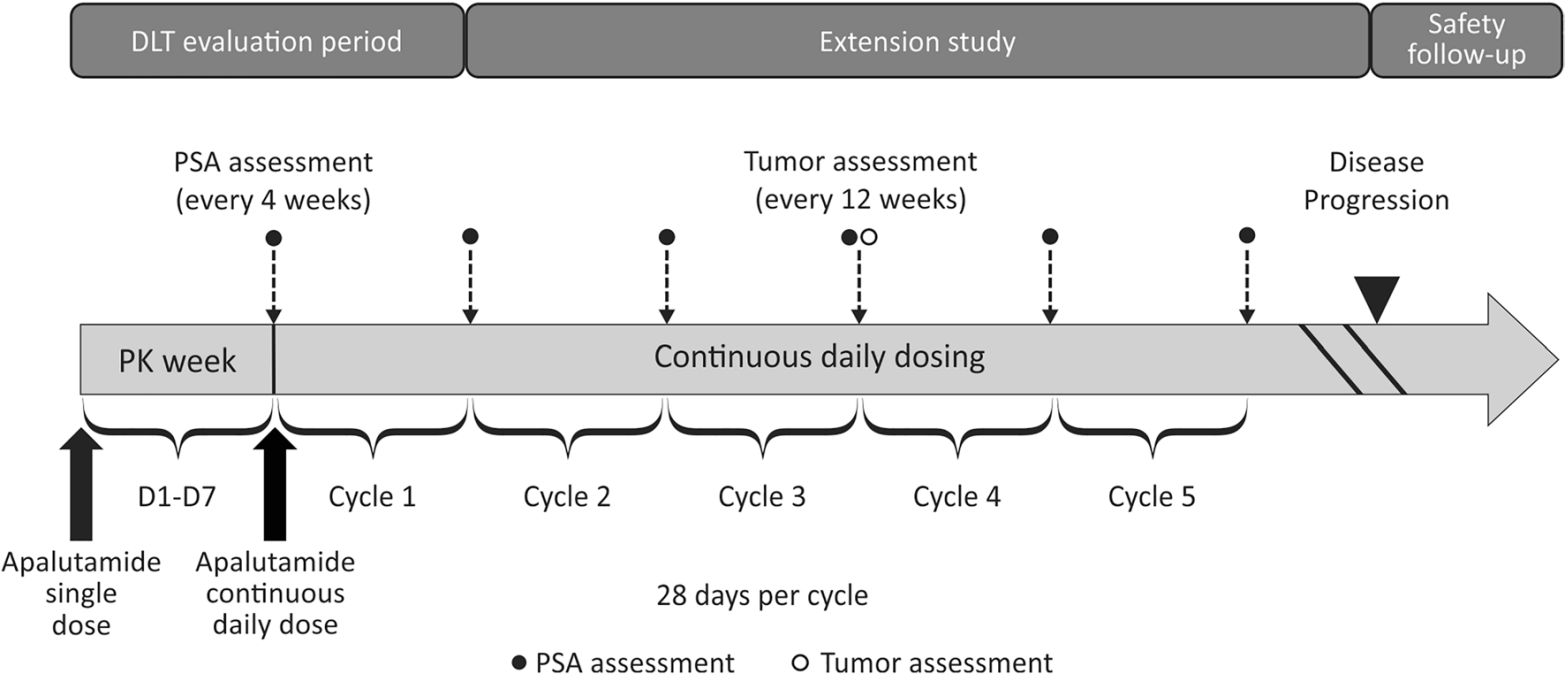
Schematic overview of the study. *D1–D7* day 1 to day 7, *DLT* dose-limiting toxicity, *PK* pharmacokinetics, *PSA* prostate-specific antigen

In this phase 1, open-label, multi-centre study, the safety, tolerability, PK, and preliminary evidence of the antitumor effects of apalutamide (240 mg, once-daily orally) was evaluated in Japanese patients with mCRPC. This study comprised 3 periods: a screening period (28 days before study treatment), a treatment period (PK week and continuous daily dosing), and a post-treatment safety follow-up period (30 days after treatment discontinuation). Dose-limiting toxicity (DLT) evaluation period was defined as the PK week (1 week) and Cycle 1 (4 weeks) (Fig. 1).

In PK week, patients received a single oral dose of apalutamide 240 mg on day 1. After a week, patients were reassessed for safety to check treatment-emergent adverse events (TEAEs), serious TEAEs, DLT, Grade 3 toxicities, and other safety parameters on Cycle 1 day 1 (day 8) and these safety profiles were compared with those of the phase 1 global study. If no new safety signals, no difference in the frequency or the degree of an event was reported, the safety profile was acceptable. The patients were then started on continuous daily oral therapy of apalutamide 240 mg until disease progression, unacceptable toxicity, withdrawal of consent, or death, whichever occurred first. If any DLT was reported, the treatment was stopped and restarted only after the toxicities were resolved to ≤ Grade 1 or baseline. After recovery, the dose was to be reduced accordingly (level 1: 180 mg/day, level 2: 120 mg/day) and restarted in accordance with the original schedule. Patients were to be withdrawn from the study if > 2 dose-level reduction was required or if the treatment did not restart within 28 days after occurrence of toxicity in principle. Intra-patient dose escalation was not allowed.

The study protocol and amendments were approved by the local Institutional Review Board and the study was conducted in accordance with the ethical principles outlined in the Declaration of Helsinki. The study was consistent with the International Conference on Harmonization, Good Clinical Practice guidelines, and applicable regulatory requirements. Written informed consent was obtained from all patients to participate in the study. The study was registered at ClincalTrials.gov: NCT02162836.

### Study evaluation

#### Safety and tolerability evaluation

Safety assessments included results of TEAEs, clinical laboratory tests, vital signs (blood pressure, pulse rate, and body temperature) and Eastern Cooperative Oncology Group (ECOG) performance status, 12-lead electrocardiograms findings, and physical examination. All TEAEs were graded using the National Cancer Institute Common Terminology Criteria for Adverse Events version 4.0 (NCI CTCAE v4.0).

The tolerability of apalutamide 240 mg in Japanese patients was evaluated based on the incidence of DLT (Online Resource 1). A study evaluation team (SET) was involved for final evaluation of toxicities based on DLT criteria, and for final decision on the safety and tolerability of apalutamide (240 mg). DLTs were assessed during the PK week and Cycle 1. Safety and tolerability of apalutamide 240 mg in Japanese patients with mCRPC were confirmed, if ≤ 1/6 patients experienced DLT. TEAEs of special interest (seizure, skin rash, hypothyroid, fall, and fracture) were also assessed.

#### Pharmacokinetics

Venous blood samples (approximately 3 mL each) for the determination of plasma concentrations of apalutamide and JNJ-56142060 (active metabolite) were collected and analyzed using a validated, specific, and sensitive liquid chromatography/mass spectrometry/mass spectrometry method. The plasma PK parameters for apalutamide, *C*_max_ (maximum observed plasma concentration), *t*_max_ (time to reach the maximum observed plasma concentration), *t*_1/2*λ*_ [elimination half-life associated with the terminal slope (*λz*) of the semi-logarithmic drug concentration–time curve, calculated as 0.693/*λz*], and AUC_0–24_ (area under the plasma concentration–time curve from time 0–24 h postdose) were determined via non-compartmental analysis with Phoenix WinNonlin (Version 6.4). Parameters AUC_last_ [AUC from time 0 to time the last observed quantifiable concentration (*C*_last_)] and AUC_∞_ (AUC from time 0 to infinite time, calculated as the sum of AUC_last_ and *C*_last/*λz*_) were determined after a single oral administration (PK week). The parameters determined only after multiple oral administrations (Cycle 1 and later) were accumulation (Acc) index, effective half-life (EHL), percentage peak to trough fluctuation (PTF)%, and *C*_trough_ (trough plasma concentration just before dose). The following plasma PK parameters were determined for the metabolite JNJ-56142060: *C*_max,_*t*_max,_ AUC_0–24,_*C*_trough_, Acc index, and metabolite-to-parent ratio (MPR).

#### Antitumour efficacy assessment

Assessment of antitumor activity of apalutamide was based on percentage change in serum PSA level from baseline reported at week 12 (or after treatment discontinuation, whichever occurred first). The maximal change was also reported for each patient using waterfall plots, at any time during the study. Serum PSA level was measured at screening visit, day 1 of PK week, day 1 of every cycle, and the end of treatment (EOT) visit by a central laboratory. Soft-tissue disease (measured by CT scans) and bone disease (evaluated radionuclide bone scans) were assessed at screening visit, and day 1 of every 3 cycles (e.g., Cycles 4, 7, 10, and so on), and the EOT visit.

#### Criteria for disease progression

Apalutamide was continued until both PSA progression and radiographic progression were documented, or until clinical progression such as a skeletal-related event, or until the treating physician decided to initiate a new systemic anticancer therapy. PSA progression was defined as PSA of ≥ 25% and ≥ 2 ng/mL above the nadir, which was confirmed by a second value obtained ≥ 3 weeks later or defined as PSA of ≥ 25% and ≥ 2 ng/mL above baseline after week 12. Visible soft-tissue tumors [measurable (target) or non-measurable (non-target) lesions] were evaluated using RECIST Version 1.1. Radiographic progression was defined as soft-tissue progression evaluated by RECIST Version 1.1, which was confirmed on repeat imaging ≥ 6 weeks later or by bone progression by Prostate Cancer Clinical Trials Working Group (PCWG) 2 [8].

Symptomatic clinical progression was defined by development of a skeletal-related event (pathologic fracture, spinal-cord compression, and need for surgery on bone or radiotherapy to bone) or progression of pain (decision was based on principal investigator’s judgement; and as pain status was an exploratory endpoint, it was not checked on regular basis). The appearance of ≥ 2 new lesions on day 1 of Cycle 4 visit with ≥ 2 additional new lesions on confirmatory bone scan was considered as disease progression.

### Statistical methods

Six patients were to be enrolled in the study to evaluate the tolerability of apalutamide at 240 mg. However, sample size was not based on statistical considerations. All-treated population set was defined as all patients who received ≥ 1 dose of apalutamide, PK analysis population set was defined as all patients who received ≥ 1 dose of apalutamide and had ≥ 1 post-treatment sample collected for PK evaluation. All-treated population set was used for efficacy and safety analyses. Patients were excluded from the PK analysis if their data did not allow accurate assessment of the PK. Plasma concentrations and corresponding PK parameters for apalutamide and JNJ-56142060 were summarized using descriptive statistics.

Percentage change from baseline in serum PSA level at week 12 was summarized using descriptive statistics and presented graphically using waterfall plots. PSA and percentage change from baseline was also presented graphically using individual case plot. Time to PSA progression was summarized using descriptive statistics. The median time of PSA progression was estimated by Kaplan–Meier method.

## Results

### Patients

A total of 15 patients were screened and 6 patients of those were enrolled and treated with apalutamide at 4 sites in Japan. At the time of clinical data cutoff (15 March 2017), 5/6 patients (83.3%) discontinued treatment and 1/6 patient (16.7%) was continuing the treatment. The treatment discontinuation was primarily due to disease progression [3/6 patients (50.0%)], physician decision, and withdrawal of consent [1/6 patient (16.7%) each].

Patient demographics and baseline characteristics are shown in Table 1. Overall, the median age of the patients was 78.0 (range 70–85) years; mean (SD) body mass index (BMI) was 22.04 kg/m^2^ (0.973). All patients had an ECOG performance status of 0–1. All patients received prior hormonal therapy and none of them received chemotherapy.

**Table 1:** Demographic and baseline characteristics (all-treated population)

	Apalutamide 240 mg (*N* = 6)
Age (years), median (range)	78.0 (70–85)
BMI (kg/m^2^), mean (SD)	22.04 (0.973)
Time from initial diagnosis to first dose (months), median (range)	68.9 (9.2–132.7)
PSA at initial diagnosis (ng/mL), median (range)	112.20 (4.8–1169.3)
Extent of disease at screening, *n* (%)	
Bone	4 (66.7)
Bone only	1 (16.7)
Soft tisssue	5 (83.3)
Lymph node	3 (50.0)
Prostate mass	4 (66.7)
Other	2 (33.3)
Tumor stage at initial diagnosis, *n* (%)	
T2, T2a, T2b, T2c	3 (50.0)
T3, T3a, T3b	2 (33.3)
T4	1 (16.7)
Metastasis stage at initial diagnosis, *n* (%)	
M0	4 (66.7)
M1, M1a, M1b, M1c	2 (33.3)
ECOG performance status score at screening, *n* (%)	
0	5 (83.3)
1	1 (16.7)
Gleason score at initial diagnosis, *n* (%)	
< 7	1 (16.7)
7	1 (16.7)
≥ 8	4 (66.7)
Prior therapy, *n* (%)	
Hormonal therapy	6 (100.0)
Chemotherapy	0
Radiotherapy	1 (16.7)
Surgery	2 (33.3)
Prostate	1 (16.7)
Orchiectomy	1 (16.7)
Other	2 (33.3)

*BMI* body mass index, *ECOG* Eastern Cooperative Oncology Group, *PSA* prostate-specific antigen, *SD* standard deviation

### Treatment compliance and extent of exposure

Overall, median (range) of drug compliance was 98.3% (69.3–100.0%). Of total, 3/6 patients (50%) achieved 100% compliance, 2/6 patients (33.3%) achieved ≥ 80 to ≤ 100% compliance and 1 patient (16.7%) achieved < 80% compliance. The median duration of study agent from day 1 Cycle 1 (except PK week) was 169.0 (range 74–869) days. At the time of clinical cutoff, all the 6 patients had received at least 3 cycles and 2 patients (33.3%) received at least 30 cycles of study agent. The median average daily dose of study agent was 236.0 (range 170.3–240.0) mg/day.

### Safety

#### Adverse events

All patients experienced at least one TEAE. The most frequently reported TEAEs (> 1 patient) were abdominal discomfort, nasopharyngitis, dysgeusia, rash, and hot flush [2/6 patients (33.3%) each] (Table 2). The drug-related TEAEs reported in > 1 patient were abdominal discomfort, dysgeusia, and hot flush [2/6 patients (33.3%) each]. One patient (16.7%) reported serious TEAE (Grade 3 spinal-cord compression) and, therefore, discontinued the study agent. A total of 2/6 patients (33.3%) reported Grade 3 TEAEs [spinal-cord compression and renal disorder, 1/6 patients (16.7%) each]. However, at data cutoff, renal disorder was resolved, while the event of spinal-cord compression was resolving. No death and no DLTs were reported. Dose interruption was reported in 2/6 patients (33.3%) due to TEAEs [Grade 2 rash generalized and Grade 1 toxic skin eruption, 1/6 patients (16.7%) each]. Three patients (50.0%) were reported with TEAEs of special interest (rash in 2 patients and rib fracture in 1 patient). The Grade 1 rash of the special interest TEAE resolved in both the patients without any action on dose of the study agent; however, it reoccurred in one patient as Grade 2 rash generalized on day 55. The event of rash generalized resolved on day 74 and reoccurred on day 83 with similar grade. It was reported to be resolving after dose interruption at time of clinical cutoff. Grade 1 rib fracture was observed in 1/6 patient (16.7%), no action on dose of the study agent was taken and the event was not recovered at clinical cutoff date. No seizure, hypothyroid, or fall was reported (Table 2).

**Table 2:** Overview of treatment-emergent adverse events through week 24 (all-treated population)

	Apalutamide 240 mg (*N* = 6) *N* (%)
Patients with ≥ 1 TEAEs	6 (100)
Serious TEAEs	1 (16.7)
Grade 3 or higher TEAEs	2 (33.3)
Spinal-cord compression	1 (16.7)
Renal disorder	1 (16.7)
TEAEs leading to discontinuation of study agent	1 (16.7)
Dose-limiting toxicity	0
Death	0
TEAEs ≥ 2 patients	
Abdominal discomfort	2 (33.3)
Nasopharyngitis	2 (33.3)
Dysgeusia	2 (33.3)
Rash	2 (33.3)
Hot flush	2 (33.3)
TEAEs of special interest^a^	
Rash	2 (33.3)
Rash generalized	1 (16.7)
Rib fracture	1 (16.7)

*TEAEs* treatment-emergent adverse events

^a^TEAEs of special interest includes seizure, skin rash, hypothyroid, fall, and fracture

#### Clinical laboratory evaluation

The Grade 3 hematology laboratory abnormalities were lymphocyte count decreased and lymphocyte count increased [1/6 patient (16.7%) each].

### Pharmacokinetic assessment

PK analysis was performed in all the six patients; however, one patient was excluded from the descriptive statistical analysis due to vomiting after the first dose. Nonetheless, after multiple doses, parameters not affected by vomiting were included. A steady state of apalutamide was reached approximately by day 22 of Cycle 1 (week 4), as indicated by the mean trough concentration–time profile (data not shown). The median *t*_max_ of apalutamide was reached at 1.58 h after single-dose administration and 1.44 h after multiple-dose administration. The mean *C*_max_ of apalutamide was approximately twofold higher after multiple-dose administration (7.57 μg/mL at Cycle 1 day 22) compared with single-dose administration (3.88 μg/mL at PK week day 1). On Cycle 1, day 22, mean Acc index based on AUC_0–24_ of apalutamide was 3.55. After single-dose administration, individual *t*_1/2*λ*_ of apalutamide ranged from 110 to 231 h; however, the data should be cautiously interpreted owing to the short sampling period (up to 168 h). After multiple-dose administration, mean EHL was 50.2 h and mean PTF% was 177% for apalutamide. A steady state of JNJ-56142060 was reached approximately by day 1 of Cycle 2 (week 5) (data not shown). The mean *C*_max_ of JNJ-56142060 was approximately 19-fold higher after multiple-dose administration (7.11 μg/mL at Cycle 1 day 22) compared with single-dose administration (0.366 μg/mL at PK week day 1). Mean Acc index based on AUC_0–24_ of JNJ-561402060 was 57.3. Mean MPR after single-dose administration and multiple-dose administration for AUC_0–24_ was 0.0814 and 1.24, respectively (Table 3).

**Table 3:** Summary of PK parameters of apalutamide and its metabolite JNJ-56142060 in plasma (PK analysis population)

	Apalutamide 240 mg		JNJ-56142060	
	PK week day 1 (*N* = 5)	Cycle 1 day 22 (*N* = 6)	PK week day 1 (*N* = 5)	Cycle 1 day 22 (*N* = 6)
*C*_max_ (μg/mL)				
Mean (SD)	3.88 (0.793)	7.57 (1.19)	0.366 (0.0751)	7.11 (0.551)
CV%	20.4	15.7	20.5	7.80
*t*_max_ (h)				
Median	1.58	1.44	168	3.68
Range	(1.00–2.05)	(0.950–4.00)	(95.5–168)	(0.00–23.8)
AUC_0–24_ (h μg/mL)				
Mean (SD)	33.6 (4.78)	122 (17.5)	2.69 (0.307)	150 (15.6)
CV%	14.2	14.3	11.4	10.4
AUC_last_ (h μg/mL)				
Mean (SD)	116 (14.1)	–	45.4 (8.59)	–
CV%	12.1	–	18.9	–
Acc index				
Mean (SD)	–	3.55 (0.139)^a^	–	57.3 (6.41)^a^
CV%	–	3.90	–	11.2
EHL (h)				
Mean (SD)	–	50.2 (2.34)^a^	–	–
CV%	–	4.70	–	–
PTF% (%)				
Mean (SD)	–	177 (23.5)	–	–
CV%	–	13.2	–	–
MPR for *C*_max_				
Mean (SD)	–	–	0.0987 (0.0314)	0.951 (0.104)
CV%	–	–	31.8	10.9
MPR for AUC_0–24_				
Mean (SD)	–	–	0.0814 (0.0159)	1.24 (0.159)
CV%	–	–	19.6	12.8

*Acc* accumulation, *CV* coefficient of variation, *EHL* effective half-life, *MPR* metabolite-to-parent ratio, *PK* pharmacokinetics, *PTF* peak to trough fluctuation, *SD* standard deviation

^a^*N* = 5 patients

### Efficacy assessment

**Fig. 2:**
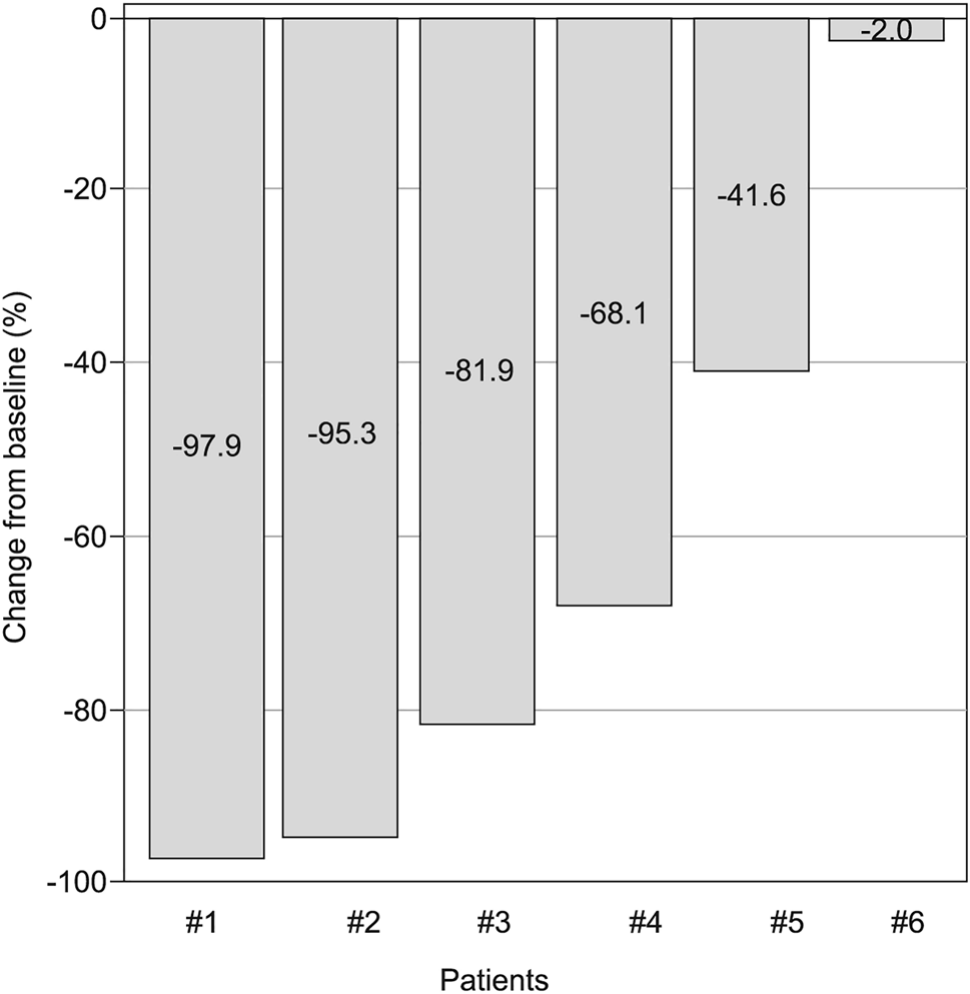
Waterfall plots of prostate-specific antigen percent change from baseline at week 12 (all-treated population)

The median serum PSA level decreased from 54.42 (range 8.92–310.11) ng/mL at baseline to 11.70 (range 0.37–47.74) ng/mL at week 12. Most of the patients [4/6 patients (66.7%)] achieved at least 50% reduction from baseline in serum PSA level at week 12 and a few patients could also achieve 90% reduction [2/6 patients (33.3%)] (Fig. 2). The median maximum reduction of percentage change from baseline in serum PSA level during treatment was − 88.3% (range − 41.6 to − 99.8%) (Table 4).

**Table 4:** Summary of prostate-specific antigen assessment (all-treated population)

	Apalutamide 240 mg (*N* = 6)
PSA (ng/mL)	
Baseline, median (range)	54.420 (8.92–310.11)
12 weeks, median (range)	11.700 (0.37–47.74)
Percent change from baseline at 12 weeks, median (range)	− 75.04 ( − 97.9 to − 2.0)
Maximum reduction from baseline, median (range)	− 29.630 ( − 295.45 to − 3.77)
Maximum reduction of percent change from baseline, median (range)	− 88.26 ( − 99.8 to − 41.6)
PSA progression	
Number of events (%)	2 (33.3)
Number of censored (%)	4 (66.7)
Time to event (months)	
Median (95% CI)	27.89 (5.82, NE)

*CI* confidence interval, *NE* not evaluable, *PSA* prostate-specific antigen

At clinical cutoff, 4/6 patients showed progression of disease [PSA progression and radiographic progression: 2/6 patients (33.3%), clinical progression: 1/6 patients (16.7%), disease progression (PSA was reported to be > 40 and no pain progression was observed) confirmed by the investigator: 1/6 patients (16.7%)]. One patient with radiographic disease progression had bone metastases and the other patient had metastatic progression of prostate mass at periaortic lymph node and both sides of internal iliac artery lymph node. The patient who did not meet predefined progression of disease criteria showed increase in serum PSA level with no pain progression.

## Discussion

This open-label study assessed the safety, tolerability, PK, and antitumor effects of apalutamide after single and multiple doses in Japanese men with mCRPC. Apalutamide administered at 240 mg once-daily dose showed manageable toxicity and overall TEAEs were consistent with the safety profile reported in global phase 1 studies of apalutamide [9–12]. No deaths, drug-related serious TEAEs, any Grade 4 or higher TEAEs, DLTs or seizure were reported in this study. Two patients required dose interruptions due to TEAEs.

After single-dose administration of apalutamide 240 mg, mean *C*_max_ and AUC_0–24_ (3.88 μg/mL and 33.6 μg h/mL, respectively) in Japanese patients with mCRPC were higher than *C*_max_ and AUC_0–24_ (2.97 μg/mL and 21.9 μg h/mL, respectively; unpublished data) observed in non-Japanese patients with mCRPC (*N* = 3). However, similar mean *C*_max_ (7.57 vs 7.55 μg/mL) and AUC_0–24_ (122 vs 127 μg h/mL) were observed in Japanese vs non-Japanese patients with mCRPC, after multiple-dose administration of apalutamide 240 mg [9]. Moreover, mean MPR for AUC_0–24_ in Japanese and non-Japanese patients with mCRPC were also comparable (1.24 vs 1.09; unpublished data).

The efficacy results of this phase 1 study demonstrated durable decline in PSA with apalutamide 240 mg once-daily dose, suggesting that the drug was effective and showed comparable trend with the result of global phase 1/2 study (NCT01171898) [7, 12]. In this study, a ≥ 50% reduction in serum PSA level from baseline was confirmed in 4/6 patients (66.7%) at week 12. Apalutamide 240 mg once-daily dose was approved in US [13] and Japan [14] for nmCRPC following positive results from the phase 3 SPARTAN study, where apalutamide confirmed significant improvements in metastasis-free survival (*P* < 0.001) and progression-free survival (*P* < 0.001) as compared to placebo [15]. Apalutamide also showed a very good risk–benefit balance in phase 3 TITAN study involving Japanese patients with metastatic castration-sensitive prostate cancer (mCSPC), where apalutamide confirmed significant improvements in radiographic progression-free survival (*P* < 0.001) and overall survival (*P* < 0.005) as compared to placebo [16].

The small sample size of the study restrains reliable demonstration of the efficacy. However, the small sample size is commonly observed in early development studies and is sufficient to allow clinical judgment of safety and tolerability, and assessment of PK profile. Furthermore, as this was a single-dose study, it was difficult to determine dose proportionality.

In conclusion, apalutamide 240 mg was well-tolerated without any DLT or any new safety signals with a favourable efficacy in Japanese mCRPC patients. In addition, the pharmacokinetic profile of apalutamide 240 mg once-daily in Japanese mCRPC patients was consistent with the established profile in the non-Japanese population. Thus, apalutamide 240 mg (once-daily, orally) was ascertained to be an adequate dosage regimen in Japanese mCRPC patients.

## Electronic supplementary material

Below is the link to the electronic supplementary material.

Supplementary file1 (DOCX 20 kb)
