# Supplementary material for: An open-label, phase 1 study of androgen receptor antagonist, apalutamide in Japanese patients with metastatic castration-resistant prostate cancer
Source: Int J Clin Oncol. 2019 Aug 24;24(12):1596–604. doi: 10.1007/s10147-019-01526-7 (PMC6861345; doi:10.1007/s10147-019-01526-7)
Supplement: Supplementary file 1 — Supplementary file1 (DOCX 20 kb) [file 10147_2019_1526_MOESM1_ESM.docx]

**An open-label, phase 1 study of androgen receptor antagonist, apalutamide in Japanese patients with metastatic castration-resistant prostate cancer**

**Journal name: International Journal of Clinical Oncology**

Tsuchiya Tomohiro^1^; Imanaka Keiichiro^2^; Iwaki Yuki^2^; Oyama Ryo^2^; Hashine Katsuyoshi^3^; Yamaguchi Akito^4^; Hiroji Uemura^5*^

^1^Gifu University Hospital

^2^Janssen Pharmaceutical K.K., Tokyo, Japan

^3^National Hospital Organization Shikoku Cancer Centre

^4^Harasanshin Hospital

^5^Yokohama City University Medical Centre

***Corresponding author:**

Dr. Hiroji Uemura

**Address**: 4-57, Urafune-cho, Minami-ku, Yokohama, 232-0024

**E-mail**: [hu0428@yokohama-cu.ac.jp](mailto:hu0428@yokohama-cu.ac.jp)

**Tel no:** +81-045-261-5656

**Fax no:** +81-045-253-1962

# **Online Resource 1 Definition of dose-limiting toxicity**

| 1 | Any Grade 3 or 4 non-hematologic toxicity |
| --- | --- |
| 2 | Grade 4 neutropenia for ≥5 consecutive days |
| 3 | Grade 4 thrombocytopenia or Grade 3 thrombocytopenia with bleeding that required platelet transfusion |
| 4 | Any other Grade 4 hematologic toxicity of ≥5 days |
| 5 | Any grade treatment-related seizure |
